# Effects of Dietary Monoglyceride and Diglyceride Supplementation on the Performance, Milk Composition, and Immune Status of Sows During Late Gestation and Lactation

**DOI:** 10.3389/fvets.2021.714068

**Published:** 2021-08-16

**Authors:** Hanqing Song, Wei Chai, Fei Yang, Man Ren, Fang Chen, Wutai Guan, Shihai Zhang

**Affiliations:** ^1^Guangdong Provincial Key Laboratory of Animal Nutrition Control, College of Animal Science, South China Agricultural University, Guangzhou, China; ^2^College of Animal Science, Anhui Science and Technology University, Fengyang, China; ^3^Anhui Provincial Key Laboratory of Animal Nutritional Regulation and Health, Fengyang, China; ^4^National Engineering Research Center for Breeding Swine Industry, College of Animal Science, South China Agricultural University, Guangzhou, China

**Keywords:** sow, monoglyceride and diglyceride, reproductive performance, milk composition, soybean lipids

## Abstract

Monoglyceride and diglyceride (MGDG) have antiviral and antibacterial properties and act as emulsifiers to increase dietary lipid digestibility. The primary aim of this trial was to investigate the effects of dietary MGDG supplementation on the reproductive performance and health status of sows during late gestation and lactation. One hundred sows (Landrace × Large White, mean parity of 4.59) were randomly allocated to groups receiving two different diets with 4% soybean lipids or 4% MGDG from day 85 of gestation to day 21 of lactation. Milk samples were collected on the day of farrowing (colostrum) and on day 14 of lactation, and blood samples were collected from the sows on days 0, 14, and 21 of lactation. Compared with control sows, sows fed MGDG showed no significant differences in reproductive performance (*P* > 0.05), but sow back fat thickness loss decreased during lactation (*P* < 0.05). There was a significant decrease in TNF-α concentrations in colostrum in the MGDG-supplemented sows compared with that in the soybean lipid-supplemented sows (*P* < 0.05). Dietary MGDG supplementation decreased sow plasma IL-8 concentrations on day 0 of lactation and IL-18 concentrations on days 14 and 21 of lactation (*P* < 0.05). Administration of MGDG increased the glucose and total cholesterol concentrations in sow plasma on day 14 and day 21, respectively (*P* < 0.05). The findings in this study suggest that MGDG supplementation could be effective in reducing back fat loss, decreasing inflammatory factor levels, and controlling total cholesterol (TCHO) concentrations during lactation.

## Introduction

Monoglyceride (MG) and diglyceride (DG) are critical hydrolyzed products of dietary triglycerides. Under normal physiological conditions, the hydrolysis of dietary triglycerides starts from the stomach, and 5–40% of triglycerides are hydrolyzed by gastric lipase (1). These undigested triglycerides will be further hydrolyzed in the duodenum, which produces glycerols, MGs, DGs, and free fatty acids (2, 3). After digestion, MGs, free fatty acids, and other lipids are associated with bile salts to form micelles for absorption.

MG and DG not only act as intermediate metabolites during triglyceride digestion but are also considered excellent water-in-oil (W/O)-type emulsifiers due to their specific amphiphilic molecular structures (hydrophilic glyceryl radicals and lipophilic alkyl radicals). Emulsifying agents have been found to promote the incorporation of fatty acids into micelles and to increase the digestibility of fat when added to the diets of rats and chicks (4, 5). The carbon chain length of fatty acids has been proposed to affect the formation of micelles (6). It is more difficult for long-chain fatty acids (LCFAs) to form micelles than medium-chain fatty acids (MCFAs) and short-chain fatty acids (SCFAs). Soybean lipids are widely used in animal diets and are mainly composed of LCFAs. To enhance the digestibility of dietary lipids, supplementation with emulsifiers has been reported as an efficient way to enhance the formation of micelles (7). Currently, a cocktail of monoglycerides and diglycerides (MGDG) is widely used as an emulsifier and/or stabilizer in the food industry (8, 9). Therefore, monoglycerides and diglycerides (MGDGs) may be efficiently utilized as energy sources to relieve weight loss and improve animal production performance.

In addition, monoglycerides have been known for a long time to have strong antiviral and antibacterial properties (10, 11). Monolinolein is reported as a more efficient antibacterial compound than its corresponding fatty acids (12). The sites of action on bacteria are the cell wall and cell membrane. Monoglycerides are reported to be incorporated into the lipid membrane and cause destabilization of the bilayer (13). For instance, monoglycerides have antibacterial activities against *Helicobacter pylori* (14). Both monolinolein and monolinolenin have antibacterial activity against Group B *Streptococcus* (12). Intriguingly, the antimicrobial and antiviral activities of MG and DG are partially dependent on the nature of fatty acids (15).

Sufficient energy intake and less bacterial infection are critical for sows to maintain their productive performance during late gestation and lactation. Previously, it has been reported that lipids can be used to increase the energy available to the sow during gestation and lactation. The addition of lipids to sow diets attenuates weight loss and improves the weight gain and survival of piglets (16, 17). Because MGDG is easily digested and exhibits antibacterial properties, we propose that MGDGs may have beneficial effects on the health status of sows. The aim of this study was to investigate the effects of dietary MGDG supplementation on sow performance, back fat thickness, milk composition, and inflammatory factors during late pregnancy and lactation.

## Materials and Methods

### Animals and Experimental Design

The experimental procedures followed a protocol approved by the South China Agricultural University Animal Care and Use Committee (No. 20110107-1, Guangzhou, China). One hundred multiparous sows (Large White × Landrace, mean parity of 4.59) were selected for the study. The sows were categorized by parity, backfat thickness, and historical reproductive performance before being randomly assigned to two dietary treatment groups, a control group (*n* = 50) and an MGDG group (*n* = 50), to ensure that these characteristics were balanced between treatments.

This trial was conducted from gestation day (G) 85 to lactation day (L) 21 at a commercial pig farm. The control group diet included 4% soybean lipids, and the MGDG group diet included 4% MGDGs (containing 40% monoglycerides and 50% diglycerides; Guangzhou Jiadele Nutrition Technology Co., Ltd., Guangzhou, China). From days 85 to 109 of gestation, all sows were managed in the same gestation facility, which was a semi-open building. On G109, all sows were moved to farrowing crates. During the entire experimental period, the sows were assigned to a pad/fan-cooled farrowing house (for active cooling, AC), and the sows within each dietary treatment were fed the same experimental diet during both late gestation and lactation.

### Diets and Management

Each diet was formulated to meet or exceed the nutrient requirements of late gestating and lactating sows (18). Table 1 shows the diet compositions and nutrient levels. In the experimental diets, the 4% lipid source was either soy lipids or MGDGs. The fatty acid composition of the supplemental fat sources was analyzed by gas chromatography (GC-2010 Pro, SHIMADZU Corporation, Kyoto, Japan). Mono- and diglycerides were measured according to the AOAC official method [(19), 993.18]. The fatty acid profiles of soy lipids and MGDGs are described in Table 2.

**Table 1:** Ingredients and chemical compositions of the diets (as-fed basis).

**Item**	**Control diet**	**MGDG diet**
Ingredient (%)		
Corn	43.8	43.8
Soybean meal, 43.0% CP	24.00	24.00
Barley, 11.33% CP	10.00	10.00
Wheat bran, 15.7% CP	6.00	6.00
Wheat flour, 15.3% CP	5.00	5.00
Fish meal, 62.8% CP	2.00	2.00
Soy lipids	4.00	
MGDG		4.00
Dicalcium phosphate	1.00	1.00
Vitamin and mineral premix^a^	4.00	4.00
Mold inhibitor	0.20	0.20
Total	100.00	100.00
Nutrient composition, unit		
DE, Mcal/kg	14.27	14.65
ME, Mcal /kg	13.72	14.27
NE, Mcal /kg	10.31	10.63
CP, %	17.93	17.93
EE, %	6.54	6.54
CF, %	3.31	3.31
Ca, %	1.00	1.00
Total P, %	0.74	0.74
Available P, %	0.50	0.50
Lys	1.04	1.04
Met + Cys	0.62	0.62
Thr	0.68	0.68
Trp	0.22	0.22
Digestible Lys, %	0.91	0.91
Digestible Met + Cys, %	0.51	0.51
Digestible Thr, %	0.57	0.57
Digestible Trp, %	0.19	0.19

a *Per kilogram of complete diet, the vitamin and mineral premix supplied the following: vitamin A, 10,000 IU; vitamin D3, 3,600 IU; vitamin E, 100 IU; vitamin K_3_, 7.2 mg; vitamin B_1_, 3 mg; riboflavin, 10.8 mg; vitamin B_6_ 5.4 mg; vitamin B_12_, 0.06 mg; D-pantothenic acid, 36.0 mg; niacin, 60.0 mg; folic acid, 6 mg; biotin, 0.6 mg; copper, 10 mg (as CuSO_4_.5H_2_O); iron, 80 mg (as FeSO_4_. H_2_O); manganese, 30.0 mg (as MnSO_4_); zinc, 80.0 mg (as ZnSO_4_); selenium, 0.15 mg (as Na_2_SeO_3_); iodine, 0.14 mg [as Ca (IO_3_) _2_]; and cobalt, 0.1 mg (as CoCl_2_.6H_2_O)*.

**Table 2:** Fatty acid composition of the Soybean Lipids MGDGs.

**Composition**	**Soybean lipids**	**MGDGs**
C16:0	11.96	4.16
C18:1	23.83	2.54
C18:2	52.71	25.17
C18:3	4.81	0.76
C18:0	4.02	6.37
C16:0&C16:0	–	0.76
C16:0&C18:0	–	4.02
C16:0&C18:2	–	6.49
C18:1&C18:1-2	–	16.42
C18:2&C18:2-3	–	22.43
Others	2.67	0.98

The experiment began on day 85 of pregnancy. At this time, the sows were individually housed in gestation crates with a solid concrete floor and had free access to drinking water. Approximately 1 week before parturition, the sows were moved into the environmentally controlled farrowing house and individually housed in farrowing crates (2.2 × 2.4 × 1.5 m^3^) with a solid concrete floor and a piglet creep area with a heating lamp. The sows and the piglets had free access to water.

During gestion, the sows were fed 3.0 kg/d. The sows were not fed on the day of farrowing. After farrowing, the feed supply was increased by 1 kg/d until day 3 and then by 0.5 kg/d until day 6 of lactation. Afterward, the animals were allowed *ad libitum* consumption of the lactation diet, which was adjusted for each sow depending on daily intake. No creep feed was offered to piglets during the experiment. Piglets were cross fostered in the same dietary treatment within 24 h of birth so that piglets of similar weight were brought together. The litter size was adjusted and maintained at 10–12 piglets per sow.

### Data and Sample Collection

#### Sow and Litter Performance

At farrowing, the total number of piglets [the numbers of live, weak (BW < 0.8 kg), stillborn, and mummified piglets] and the litter weights were recorded. At weaning, the number of pigs weaned and the litter weight were measured.

#### Sow Back Fat Thickness

The back fat thickness of the sow at the P2 point (6.5 cm from the middle line of the last rib) was measured using a real-time ultrasound facility (Renco Lean-meter, Renco Corporation, Minnesota, USA) on days 0 and 14 of lactation.

#### Blood Sampling

At farrowing, on L14 and at weaning, 10 mL blood samples were taken from 10 sows (near the average BW and parity in each treatment group) by ear venipuncture using heparinized Vacutainer tubes (Sanli Medical Technology Development Co., Ltd., Hunan, China). Plasma was harvested after centrifugation at 3,000 × g for 10 min. After collection, each plasma sample was divided into three 0.5 mL samples (pipetted into 1 mL frozen tubes and immediately frozen in liquid nitrogen for immunoglobulin analysis) and a 2 mL sample (transferred to a 4 mL centrifuge tube and then stored at −80°C for cytokine and blood parameter analysis).

#### Colostrum and Milk Sampling

Colostrum and milk samples were taken from 10 sows (near the average BW and parity in each treatment group). Colostrum was sampled by hand expression from functional glands within 12 h post-partum without oxytocin injection. Fourteen-day milk was sampled after intramuscular injection of 20 IU oxytocin (Jiangxi Huiqifeng Bio-Technique Co., Ltd., Jiangxi, China).

Approximately 30 mL of sample was collected each time. After collection, the colostrum or milk was divided into two 15 mL centrifuge tubes and stored at −80°C for nutritional composition, immunoglobulin, and cytokine analyses.

### Chemical Analysis

#### Colostrum and Milk Composition

The colostrum and milk samples were tested for solids-not-fat, fat, protein, and lactose using a fully automated milk analyzer (ULTRAMILER-UL40AC, Hangzhou Ultrasun Technologies Co., Ltd., Zhejiang, China).

#### Immunoglobulin Concentrations

The immunoglobulin A (IgA), immunoglobulin G (IgG), and immunoglobulin M (IgM) concentrations in colostrum and milk were analyzed by ELISA using pig immunoglobulin-specific kits (CUSABIO Biotech Company, Wuhan, China). Prior to analysis, the lipids in colostrum and milk were removed by centrifugation at 3,000 × g and 4°C for 20 min according to a method described previously (20). The ELISA procedure was as follows: (1) A blank well was prepared without any solution. (2) Fifty microliters of a standard or sample was added to each well. (3) Fifty microliters of HRP-conjugate was added to each well (not to the blank well). (4) The wells were incubated for 40 min at 37°C. (5) The wells were aspirated and washed 5 times. (6) Ninety microliters of TMB substrate was added to each well. The wells were incubated for 20 min at 37°C. (7) Fifty microliters of stop solution was added to each well. The wells were measured with a plate reader at 450 nm within 5 min.

#### Cytokine Concentrations

Tumor necrosis factor α (TNF-α), interleukin (IL)-8, and IL-18 levels in sow plasma, colostrum and milk were determined with ELISA kits (CUSABIO Biological Engineering Co., Ltd., Wuhan, China). The ELISA procedure was as follows: (1) One hundred microliters of a standard or sample was added to each well and incubated for 2 h at 37°C. (2) The liquid of each well was removed without washing. (3) Then, 100 μL of biotin-antibody (1x) was added to each well and incubated for 1 h at 37°C. (4) The wells were aspirated and washed 3 times. (5) Then, 100 μl of HRP-avidin was added to each well and incubated for 1 h at 37°C. (6) The wells were aspirated and washed 5 times. (7) Ninety microliters of TMB substrate was added to each well and incubated for 20 min at 37°C. (8) Fifty microliters of stop solution was added to each well. The wells were measured with a plate reader at 450 nm within 5 min.

#### Blood Parameter Concentrations

The concentrations of glucose (GLU), plasma urea nitrogen (PUN), triglycerides (TGs), total cholesterol (TCHO), high-density lipoprotein cholesterol (HDL-C), and low-density lipoprotein cholesterol (LDL-C) in plasma were determined with commercial kits (Nanjing Jiancheng Bioengineering Institute, Nanjing, China). The procedure was as follows: (1) First, 2.5 μl of distilled water was added to the blank well. (2) Then, 2.5 μl of a standard or sample was added to each well. (3) A total of 250 μl of TMB substrate was added to each well. (4) The wells were incubated for 10 min at 37°C. (5) The wells were measured with a plate reader at 510 within 5 min.

### Statistical Analysis

Reproductive and lactation performance, back fat thickness, and colostrum and milk composition were analyzed by independent-sample Student's *t*-tests using SPSS 22.0 (IBM-SPSS Inc., Chicago, Illinois, USA) after checking for normality and homogeneity of variance according to the Shapiro–Wilk and Levene tests, respectively. Plasma cytokine concentration and plasma constituent concentration data were analyzed using the mixed procedure of SAS with repeated measures. The model included diet (control vs. MGDG), parity number, and timepoint (defined as a repeated measure) and their interaction as fixed effects and a random effect. The results are expressed as the means and standard error means (SEMs). The effects of treatment on the presence or absence of estrus after weaning were evaluated using chi-square analysis (21). Probability values <0.05 were considered to indicate significance, and probability values <0.10 were considered to indicate tendencies toward differences between treatments.

## Results

### Reproductive and Lactation Performance

The effects of dietary MGDG supplementation during late gestation and lactation on sow reproductive and lactation performance are shown in Table 3. The total number of piglets born, the numbers of stillborn and live-born piglets, the litter birth weights, and the individual piglet birth weights were unaffected by the treatment (*P* > 0.05; Table 3). The average daily feed intake (ADFI) and the estrus rate were not affected by MGDG supplementation (*P* > 0.05). The average daily gain (ADG), litter weight, average piglet weight, and preweaning survival during lactation were unaffected by the MGDG-supplemented diet compared with the control diet (*P* > 0.05).

**Table 3:** Effects of dietary MGDG supplementation during late gestation and lactation on sow reproductive and lactation performance.

**Parameter**	**Control**	**MGDG**	***P*-value**
No. of sows	50	50	
ADFI (day 0–day 21 of lactation), kg	5.34 ± 0.08	5.26 ± 0.10	0.634
Reproductive performance			
Total No. of pigs born/litter	11.58 ± 0.37	12.20 ± 0.36	0.771
No. of pigs born alive/litter	10.92 ± 0.37	11.64 ± 0.36	0.133
No. of stillbirths/litter	0.46 ± 0.05	0.35 ± 0.07	0.152
No. of weak pigs/litter	0.43 ± 0.12	0.70 ± 0.13	0.134
No. of mummies/litter	0.07 ± 0.01	0.11 ± 0.003	0.122
Litter birth weight, kg	16.78 ± 0.53	16.89 ± 0.50	0.442
Individual piglet weight, kg	1.56 ± 0.13	1.47 ± 0.12	0.335
Lactation performance			
No. of pigs after cross-foster	10.34 ± 0.17	10.41 ± 0.15	0.686
No. of weaning pigs	9.44 ± 0.20	9.22 ± 0.21	0.481
Preweaning survival, %	91.58 ± 1.35	88.81 ± 0.98	0.270
Individual pig weight after cross-foster, kg	1.96 ± 0.55	1.90 ± 0.54	0.907
Individual pig weight at weaning, kg	6.54 ± 0.13	6.67 ± 0.12	0.853
Litter weight after cross-foster, kg	20.02 ± 0.50	19.69 ± 0.57	0.211
Litter weight at weaning, kg	61.88 ± 1.88	61.43 ± 1.69	0.842
ADG, g/day	212.09 ± 5.80	223.31 ± 4.88	0.435

*ADFI, average daily feed in take during lactation; ADG, average daily gain. A P <0.05 indicates a significant difference among treatments*.

### Back Fat Thickness and Estrus Interval

The effects of dietary MGDG supplementation during late gestation and lactation on sow colostrum and milk composition are shown in Table 4. The loss of backfat thickness was significantly decreased after MGDG supplementation compared with the control group during lactation (*P* < 0.05; Table 4).

**Table 4:** Effects of dietary MGDG supplementation during late gestation and lactation on sow back fat thickness and estrus interval.

**Parameter**	**Control**	**MGDG**	***P*-value**
*n*	50	50	
Sow back fat			
Back fat at farrowing	18.33 ± 1.23	18.50 ± 0.72	0.905
Back fat at weaning	14.92^b^ ± 0.53	16.77^a^ ± 0.42	0.023
Back fat loss during lactation	3.41^a^ ± 0.43	2.18^b^ ± 0.29	0.029
Estrus rate within 7 days	91.49	91.30	0.795
after weaning, %			

*A P <0.05 indicates the existence of a significant difference among treatments*.

a,b *means values within a row with different letters superscripts means significantly different (P <0.05)*.

### Colostrum and Milk Composition

The effects of dietary MGDG supplementation during late gestation and lactation on sow colostrum and milk composition are shown in Table 5. No differences between the treatment and control groups were found with regard to the protein and lactose levels in colostrum and milk (*P* > 0.05; Table 5). However, the sows with dietary MGDG supplementation tended to have higher fat concentrations in their colostrum than sows fed the control diet (*P* < 0.1).

**Table 5:** Effects of dietary MGDG supplementation during late gestation and lactation on sow colostrum and milk composition.

**Parameter**	**Control**	**MGDG**	***P*-value**
*n*	10	10	
Colostrum (%)			
Non-fat-solids	20.90 ± 0.46	19.26 ± 0.92	0.109
Protein	11.33 ± 0.23	10.27 ± 0.62	0.102
Fat	4.46 ± 0.40	5.42 ± 0.32	0.087
Lactose	7.89 ± 0.19	7.23 ± 0.37	0.115
Milk (%)			
Non-fat-solids	10.35 ± 0.15	10.63 ± 0.12	0.147
Protein	3.80 ± 0.06	3.92 ± 0.04	0.123
Fat	6.93 ± 0.23	7.09 ± 0.32	0.695
Lactose	5.85 ± 0.12	5.91 ± 0.07	0.652

*A P <0.05 indicates the existence of a significant difference among treatments*.

### Immunoglobulin Concentrations

The effects of dietary MGDG supplementation during late gestation and lactation on the immunoglobulin concentrations in sow colostrum and milk are shown in Table 6. No significant treatment differences were observed in terms of IgG, IgM, and IgA levels in colostrum and milk (*P* > 0.05; Table 6).

**Table 6:** Effects of dietary MGDG supplementation during late gestation and lactation on immunoglobulin levels in sow colostrum and milk.

**Parameter**	**Control**	**MGDG**	***P*-value**
*n*	10	10	
Colostrum (mg/mL)			
IgG	11.03 ± 1.45	11.11 ± 1.06	0.858
IgA	3.12 ± 0.44	3.61 ± 0.58	0.531
IgM	2.48 ± 0.23	2.78 ± 0.31	0.326
Milk (day 14) (mg/mL)			
IgG	1.25 ± 0.12	0.91 ± 0.19	0.405
IgA	2.25 ± 0.24	2.61 ± 0.34	0.490
IgM	1.01 ± 0.09	1.18 ± 0.12	0.561

*IgG, immunoglobulin G; IgA, immunoglobulin A; IgM, immunoglobulin M. A P-threshold <0.05 indicates the existence of a significant difference among treatments*.

### Cytokine Concentrations

The effects of dietary MGDG supplementation during late gestation and lactation on the cytokine concentrations in sow colostrum, milk, and plasma are shown in Table 7. Compared with the sows fed the control diet, those fed the MGDG-supplemented diet had a lower TNF-α concentration in their colostrum (*P* < 0.05; Table 7). During the lactation period, lower cytokine concentrations in sow plasma were observed on L0 (IL-8), L14 (IL-18), and L21 (IL-18) (*P* < 0.05).

**Table 7:** Effects of dietary MGDG supplementation during late gestation and lactation on cytokine concentrations in sow colostrum, milk, and plasma.

**Parameter**	**Control**	**MGDG**	***P*-value**
*n*	10	10	
Colostrum			
IL-8, pg/mL	3592.14 ± 363.37	3142.93 ± 307.79	0.519
IL-18, pg/mL	1469.73 ± 86.98	1259.80 ± 243.93	0.332
TNF-α, ng/mL	1.37 ± 0.13^a^	0.36 ± 0.08^b^	0.026
Milk			
IL-8, pg/mL	793.09 ± 64.47	622.91 ± 80.47	0.118
IL-18, pg/mL	398.56 ± 31.12	335.89 ± 38.42	0.399
TNF-α, ng/mL	1.04 ± 0.15	0.91 ± 0.02	0.768
L0			
IL-8, pg/mL	2039.56 ± 364.63	1138.16 ± 117.11	0.047
IL-18, pg/mL	273.48 ± 38.90	238.96 ± 17.13	0.256
TNF-α, pg/mL	251.67 ± 59.46	182.16 ± 10.95	0.122
L14			
IL-8, pg/mL	975.61 ± 31.80	873.92 ± 95.87	0.615
IL-18, pg/mL	183.10 ± 26.19^a^	22.03 ± 3.00^b^	0.014
TNF-α, pg/mL	311.66 ± 79.34	266.58 ± 7.69	0.068
L21			
IL-8, pg/mL	914.05 ± 140.15	875.85 ± 89.01	0.602
IL-18, pg/mL	242.43 ± 36.31^a^	45.87 ± 5.35^b^	0.019
TNF-α, pg/mL	336.38 ± 10.93	269.62 ± 8.77	0.298

*IL-8, interleukin-8; IL-18, interleukin-18; TNF-α, tumor necrosis factor α; L0, lactation day 0; L14, lactation day 14; L21, lactation day 21. A P < 0.05 indicates the existence of a significant difference among treatments*.

a,b *means values within a row with different letters superscripts means significantly different (P < 0.05)*.

### Blood Parameter Concentrations

The effects of dietary MGDG supplementation during late gestation and lactation on blood parameters in the plasma of sows are shown in Table 8. On L14, the GLU levels in plasma were higher in MGDG-fed sows than in sows in the control group. On the day of weaning, the TCHO content in plasma was lower in the MGDG group than in the control group (*P* < 0.05; Table 8). The PUN, HDL-C, LDL-C, and TG levels were not affected by MGDG supplementation (*P* > 0.05).

**Table 8:** Effects of dietary MGDG supplementation during late gestation and lactation on sow plasma constituent concentrations.

**Item**	**Control**	**MGDG**	***P*-value**
*n*	10	10	
Day 85 of gestation (mmol/L)			
GLU	4.77 ± 0.21	4.43 ± 0.24	0.312
TG	1.87 ± 0.29	1.69 ± 0.11	0.558
TCHO	7.26 ± 0.52	8.43 ± 1.03	0.323
HDL-C	0.77 ± 0.12	0.80 ± 0.07	0.794
LDL-C	1.11 ± 0.07	0.92 ± 0.09	0.104
PUN	7.96 ± 0.33	7.71 ± 0.51	0.684
Day of farrowing (mmol/L)			
GLU	4.80 ± 0.33	5.40 ± 0.32	0.207
TG	1.87 ± 0.41	1.81 ± 0.11	0.727
TCHO	6.07 ± 0.41	5.23 ± 0.81	0.366
HDL-C	0.86 ± 0.07	0.61 ± 0.10	0.054
LDL-C	1.30 ± 0.10	1.11 ± 0.15	0.340
PUN	7.34 ± 0.65	7.89 ± 0.41	0.444
Day 14 of lactation (mmol/L)			
GLU	4.81^b^ ± 0.32	5.93^a^ ± 0.21	0.010
TG	1.71 ± 0.14	1.52 ± 0.11	0.306
TCHO	11.59 ± 0.77	10.29 ± 1.23	0.386
HDL-C	1.60 ± 0.36	1.07 ± 0.05	0.179
LDL-C	1.99 ± 0.65	1.04 ± 0.08	0.164
PUN	11.48 ± 0.37	11.13 ± 0.0.54	0.599
Day of weaning (mmol/L)			
GLU	5.88 ± 0.35	5.15 ± 0.36	0.162
TG	1.77 ± 0.20	1.45 ± 0.21	0.288
TCHO	12.15^a^ ± 1.08	9.05^b^ ± 0.58	0.021
HDL-C	1.41 ± 0.04	1.13 ± 0.08	0.111
LDL-C	1.66 ± 0.15	1.39 ± 0.12	0.127
PUN	10.21 ± 0.61	9.14 ± 0.83	0.176

*GLU, glucose; TG, triglyceride; TCHO, total cholesterol; HDL-C, high density lipoprotein cholesterol; LDL-C, low density lipoprotein cholesterol; PUN, plasma urea nitrogen. A P-threshold <0.05 indicates the existence of significant difference among treatments*.

a,b *means values within a row with different letters superscripts means significantly different (P < 0.05)*.

## Discussion

In this study, supplementation with MGDG in a sow diet reduced back fat loss during the lactation period. To date, the effects of backfat on sow reproductive performance are still inconsistent. Some contend that backfat is not a reliable predictor of subsequent sow reproductive performance (22), but other scientists argue that there is an optimal range of backfat (15–16 mm or even at least 20 mm) for maintaining subsequent reproduction (23, 24). Increasing dietary energy levels is an effective way to reduce back fat thickness loss in sows because the requirement to mobilize their body lipids is decreased. In our experiment, the MGDG diet contained a higher level of net energy (calculated value), which could directly alleviate the loss of back fat thickness. Another possible reason is that MGDG acts as an emulsifier in the sow diet, which increases the ileal apparent digestibility of dry matter, organic matter, crude protein, and gross energy of the base diet (25). This hypothesis still needs to be confirmed in the future.

Sow milk directly affects the growth and development of piglets during lactation. Protein, lactose, and fat are major nutritional components in sow milk, accounting for 4.9, 5.1, and 7.1%, respectively (26). Amino acids are a basal component of sow milk synthesis and stimulate milk synthesis through the activation of mTORC1 (27). The addition of lipids to sow diets during late pregnancy and lactation significantly increases the amount of fat in the milk of the sows (28). In addition, dietary supplementation with emulsifiers (lysophospholipids or sodium stearoyl-2-lactylate) is also reported to increase milk fat concentration (29). However, in this study, no significant difference in milk composition was observed when sows were fed the MGDG diet.

Immunoglobulins in colostrum and milk can decrease the susceptibility of newborns to infection (26). Until now, the results regarding the effects of dietary emulsifier supplementation on milk secretary immunoglobulins have not been consistent. Wang et al. (30) found that dietary emulsifier levels linearly increased IgA and IgG concentrations in colostrum and milk. Zhao et al. (29) reported that the concentration of milk IgG was not affected by emulsifier supplementation (24, 29). In this experiment, no significant difference was observed in IgG, IgA, and IgM levels. The discrepancy in the effects of emulsifiers in different studies might be due to the different efficiencies of different studies.

In pathogen invasion, bacterial endotoxin (lipopolysaccharide, LPS) can trigger the secretion of proinflammatory cytokines, such as tumor necrosis factor-α (TNF-α) and interleukin-1β (IL-1), through the activation of the myeloid differentiation factor 88 (MyD88)/nuclear factor-kappa B (NF-κB) signaling pathway (31). These inflammatory factors can cause fever and activate T and B lymphocytes, which further trigger the secretion of other inflammatory mediators (32–34). In our trial, supplementation with MGDG in a sow diet decreased the plasma cytokine concentrations (IL-18 and IL-8). Both IL-18 and IL-8 are identified as proinflammatory cytokines. IL-18 participates in natural killer cell activation and T helper 1 (Th1) cell responses, and IL-8 mainly regulates the recruitment and activation of neutrophils during inflammation (35). The potential underlying mechanisms for MGDG-inhibited secretion of IL-18 and IL-8 are listed as follows. First, MGDG has strong antiviral and antibacterial ability, which could decrease the amount of pathogenic bacteria in the intestine and decrease intestinal inflammation (20). Second, MGDG could directly bind to GPR119 (a G protein coupled to Gs) and decrease the secretion of IL-18. Previously, activation of the GPR109A (Gi) signaling pathway was found to increase the secretion of IL-18 (36). Gs and Gi signaling inversely regulate cell biological function through the modulation of adenyl cyclase (AC) (37). Thus, it is highly possible that MGDG could decease the secretion of IL-18. Further research is still required to verify this hypothesis.

At weaning, the present study found that the concentration of TCHO in sow plasma decreased, which is in agreement with the data of Jones et al. (38). Wang et al. (30) also reported that the level of serum TCHO decreased linearly in lactating sows as dietary emulsifier supplementation increased. Cholesterol is an abundant and indispensable substance in animals that can directly reflect lipid metabolism. It plays an important role in the formation of cell membranes and in the synthesis of bile acids and vitamin D (39). During lactation, excessive metabolism of adipose tissue leads to increases in plasma lactate cholesterol levels in sows. Therefore, the lower plasma concentrations of TCHO in the MGDG group suggest that the sows fed the MGDG-supplemented diet might have used less stored body tissues to supply energy for maintenance requirements and milk production during lactation. Therefore, MGDG supplementation might have enhanced the digestion and absorption of lipids, thus increasing the energy supply to lactating sows in this study.

Collectively, this study found that dietary MGDG supplementation during late gestation and lactation improves sow body condition and alleviates inflammation. Further research is warranted to examine whether dietary MGDGs regulate the inflammation of sows through the modification of intestinal microbes.

## Data Availability Statement

The raw data supporting the conclusions of this article will be made available by the authors, without undue reservation.

## Ethics Statement

The animal study was reviewed and approved by South China Agricultural University Animal Care and Use Committee. Written informed consent was obtained from the owners for the participation of their animals in this study.

## Author Contributions

HS and WC carried out this experiment collected data and wrote the manuscript. WG and SZ designed the research and gave guidance on writing paper. SZ, FC, MR, and WG reviewed the manuscript. HS, WC, and FY helped to prepare the experiment. All authors read and approved the final manuscript.

## Conflict of Interest

The authors declare that the research was conducted in the absence of any commercial or financial relationships that could be construed as a potential conflict of interest.

## Publisher's Note

All claims expressed in this article are solely those of the authors and do not necessarily represent those of their affiliated organizations, or those of the publisher, the editors and the reviewers. Any product that may be evaluated in this article, or claim that may be made by its manufacturer, is not guaranteed or endorsed by the publisher.

## References

[1] Sams L Paume J Giallo J Carrière F. Relevant pH and lipase for in vitro models of gastric digestion. Food Funct. (2016) 7:30–45. 10.1039/C5FO00930H 26527368

[2] Armand M Pasquier B André M Borel P Senft M Peyrot J. Digestion and absorption of 2 fat emulsions with different droplet sizes in the human digestive tract. Am J Clin Nutr. (1999) 70:1096–106. 10.1093/ajcn/70.6.1096 10584056

[3] Nik AM Wright AJ Corredig M. Impact of interfacial composition on emulsion digestion and rate of lipid hydrolysis using different in vitro digestion models. Coll Surf B Biointerfaces. (2011) 83:321–30. 10.1016/j.colsurfb.2010.12.001 21194901

[4] de Smidt PC Campanero MA Trocóniz IF. Intestinal absorption of penclomedine from lipid vehicles in the conscious rat: contribution of emulsification versus digestibility. Int J Pharm. (2004) 270:109–18. 10.1016/j.ijpharm.2003.10.036 14726127

[5] Roy A Haldar S Mondal S Ghosh TK. Effects of supplemental exogenous emulsifier on performance, nutrient metabolism, and serum lipid profile in broiler chickens. Vet Med Int. (2010) 2010:262604. 10.4061/2010/262604 20671938 PMC2910456

[6] Drover VA Nguyen DV Bastie CC Darlington YF Abumrad NA Pessin JE. CD36 mediates both cellular uptake of very long chain fatty acids and their intestinal absorption in mice. J Biol Chem. (2008) 283:13108–15. 10.1074/jbc.M708086200 18332148 PMC2442355

[7] McClements DJ Jafari SM. Improving emulsion formation, stability and performance using mixed emulsifiers: a review. Adv Coll Interface Sci. (2018) 251:55–79. 10.1016/j.cis.2017.12.001 29248154

[8] Krog NJ. Food Emulsifiers and Their Chemical and Physical Properties. Food Emulsions (1997).

[9] Yasukawa T Yasunaga K. Nutritional functions of dietary diacylglycerols. J Oleo Sci. (2001) 50:427–32. 10.5650/jos.50.427

[10] Bergsson G Hilmarsson H Thormar H. Antibacterial, antiviral and antifungal activities of lipids. Lipids Essential Oils. (2011) 47. 10.1002/9780470976623.ch312169299

[11] Churchward CP Alany RG Snyder LA. Alternative antimicrobials: the properties of fatty acids and monoglycerides. Crit Rev Microbiol. (2018) 44:561–70. 10.1080/1040841X.2018.1467875 29733249

[12] Isaacs CE Litov RE Thormar H. Antimicrobial activity of lipids added to human milk, infant formula, and bovine milk. J Nutr Biochem. (1995) 6:362–6. 10.1016/0955-2863(95)80003-U 12049996

[13] Bergsson G Arnfinnsson J Steingrimsson Ó Thormar H. In vitro killing of Candida albicans by fatty acids and monoglycerides. Antimic Agents Chemother. (2001) 45:3209–12. 10.1128/AAC.45.11.3209-3212.2001 11600381 PMC90807

[14] Sun CQ O'Connor CJ Roberton AM. Antibacterial actions of fatty acids and monoglycerides against *Helicobacter pylori*. FEMS Immunol Med Microbiol. (2003) 36:9–17. 10.1016/S0928-8244(03)00008-7 12727360

[15] Cateni F Bonivento P Procida G Zacchigna M Scialino G Banfi E. Chemoenzymatic synthesis and in vitro studies on the hydrolysis of antimicrobial monoglycosyl diglycerides by pancreatic lipase. Bioorg Med Chem Lett. (2007) 17:1971–8. 10.1016/j.bmcl.2007.01.019 17270436

[16] Averette LA Odle J Monaco MH Donovan SM. Dietary fat during pregnancy and lactation increases milk fat and insulin-like growth factor I concentrations and improves neonatal growth rates in swine. J Nutr. (1999) 129:2123–9. 10.1093/jn/129.12.2123 10573538

[17] Van den Brand H Soede N Kemp B. Dietary energy source at two feeding levels during lactation of primiparous sows: II. Effects on periestrus hormone profiles and embryonal survival. J Anim Sci. (2000) 78:405–11. 10.2527/2000.782405x 10709932

[18] National Research Council. Nutrient Requirements of Swine. Washington, DC (2012).

[19] AOAC. Official Methods of Analysis of AOAC International (17th ed.). Gaithersburg, MD: AOAC International (2000).

[20] Zanello G Meurens F Serreau D Chevaleyre C Melo S Berri M. Effects of dietary yeast strains on immunoglobulin in colostrum and milk of sows. Vet Immunol Immunopathol. (2013) 152:20–7. 10.1016/j.vetimm.2012.09.023 23092748

[21] Snedecor GW. Statistical methods. Soil Sci. (1989) 51:163. 10.1097/00010694-193902000-00006

[22] Aherne F. Feeding the Gestating Sow. Swine Nutrition and Management Consultant (1999).

[23] Farmer C. The Gestating and Lactating Sow: Wageningen: Wageningen Academic Publishers (2014). 10.3920/978-90-8686-803-2

[24] Kim K Hosseindoust A Ingale S Lee S Noh H Choi Y. Effects of gestational housing on reproductive performance and behavior of sows with different backfat thickness. Asian Austr J Anim Sci. (2016) 29:142. 10.5713/ajas.14.0973 26732338 PMC4698681

[25] Dierick N Decuypere J. Influence of lipase and/or emulsifier addition on the ileal and faecal nutrient digestibility in growing pigs fed diets containing 4% animal fat. J Sci Food Agric. (2004) 84:1443–50. 10.1002/jsfa.1794

[26] Zhang S Chen F Zhang Y Lv Y Heng J Min T. Recent progress of porcine milk components and mammary gland function. J Anim Sci Biotechnol. (2018) 9:1–13. 10.1186/s40104-018-0291-8 30377527 PMC6196465

[27] Wu Z Heng J Tian M Song H Chen F Guan W. Amino acid transportation, sensing and signal transduction in the mammary gland: key molecular signalling pathways in the regulation of milk synthesis. Nutr Res Rev. (2020) 33:287–97. 10.1017/S0954422420000074 32151298

[28] Boyd R Moser B Peo E Jr Lewis A Johnson R. Effect of tallow and choline chloride addition to the diet of sows on milk composition, milk yield and preweaning pig performance. J Anim Sci. (1982) 54:1–7. 10.2527/jas1982.5411 7085489

[29] Zhao P Zhang Z Lan R Liu W Kim I. Effect of lysophospholipids in diets differing in fat contents on growth performance, nutrient digestibility, milk composition and litter performance of lactating sows. Animal. (2017) 11:984–90. 10.1017/S1751731116002238 27819219

[30] Wang Q Long S Hu J Li M Pan L Piao X. Effects of dietary lysophospholipid complex supplementation on lactation performance, and nutrient digestibility in lactating sows. Anim Feed Sci Technol. (2019) 251:56–63. 10.1016/j.anifeedsci.2018.12.009

[31] Kawai T Akira S. Signaling to NF-κB by Toll-like receptors. Trends Mol Med. (2007) 13:460–9. 10.1016/j.molmed.2007.09.002 18029230

[32] Choy EH Panayi GS. Cytokine pathways and joint inflammation in rheumatoid arthritis. New Engl J Med. (2001) 344:907–16. 10.1056/NEJM200103223441207 11259725

[33] Kaaja R Laivuori H Pulkki P Tikkanen MJ Hiilesmaa V Ylikorkala O. Is there any link between insulin resistance and inflammation in established preeclampsia? Metabolism. (2004) 53:1433–5. 10.1016/j.metabol.2004.06.009 15536597

[34] GKMDPH. Mechanisms of disease: inflammation, atherosclerosis, and coronary artery disease. N Engl J Med. (2005) 352:1685–95. 10.1056/NEJMra043430 15843671

[35] Yoshimoto T Tsutsui H Tominaga K Hoshino K Okamura H Akira S. IL-18, although antiallergic when administered with IL-12, stimulates IL-4 and histamine release by basophils. Proc Natl. Acad Sci. (1999) 96:13962–6. 10.1073/pnas.96.24.13962 10570181 PMC24173

[36] Koh A De Vadder F Kovatcheva-Datchary P Bäckhed F. From dietary fiber to host physiology: short-chain fatty acids as key bacterial metabolites. Cell. (2016) 165:1332–45. 10.1016/j.cell.2016.05.041 27259147

[37] Godinho RO Duarte T Pacini ESA. New perspectives in signaling mediated by receptors coupled to stimulatory G protein: the emerging significance of cAMP efflux and extracellular cAMP-adenosine pathway. Front Pharmacol. (2015) 6:58. 10.3389/fphar.2015.00058 25859216 PMC4373373

[38] Jones D Hancock J Harmon D Walker C. Effects of exogenous emulsifiers and fat sources on nutrient digestibility, serum lipids, and growth performance in weanling pigs. J Anim Sci. (1992) 70:3473–82. 10.2527/1992.70113473x 1459909

[39] Broeders N Knoop C Abramowicz D. Drug treatment of lipid disorders. N Engl J Med. (1999) 341:2020. 10.1056/NEJM199912233412617 10617402

